# Optimizing the Comet Assay-Based In Vitro DNA Repair Assay for Placental Tissue: A Pilot Study with Pre-Eclamptic Patients

**DOI:** 10.3390/ijms25010187

**Published:** 2023-12-22

**Authors:** Anastasiya Mircheva, Philippe Vangrieken, Salwan Al-Nasiry, Frederik-Jan van Schooten, Roger W. L. Godschalk, Sabine A. S. Langie

**Affiliations:** 1 Department of Pharmacology and Toxicology, School for Nutrition and Translational Research in Metabolism (NUTRIM), Maastricht University, P.O. Box 616, 6200 MD Maastricht, The Netherlands; a.mircheva@maastrichtuniversity.nl (A.M.); r.godschalk@maastrichtuniversity.nl (R.W.L.G.); 2 Department of Internal Medicine, School of Cardiovascular diseases (CARIM), Maastricht University, P.O. Box 616, 6200 MD Maastricht, The Netherlands; 3 Department of Obstetrics and Gynecology, Maastricht University Medical Center+, P. Debyelaan 25, 6229 HX Maastricht, The Netherlands

**Keywords:** DNA repair, BER, placenta, comet-assay, preeclampsia, oxidative stress

## Abstract

The comet assay-based in vitro DNA repair assay has become a common tool for quantifying base excision repair (BER) activity in human lymphocytes or cultured cells. Here, we optimized the protocol for studying BER in human placental tissue because the placenta is a non-invasive tissue for biomonitoring of early-life exposures, and it can be used to investigate molecular mechanisms associated with prenatal disorders. The optimal protein concentration of placental protein extracts for optimal damage recognition and incision was 2 mg protein/mL. The addition of aphidicolin did not lead to reduced non-specific incisions and was, therefore, not included in the optimized protocol. The interval between sample collection and analysis did not affect BER activity up to 70 min. Finally, this optimized protocol was tested on pre-eclamptic (PE) placental tissues (*n* = 11) and significantly lower BER activity in PE placentas compared to controls (*n* = 9) was observed. This was paralleled by a significant reduction in the expression of BER-related genes and increased DNA oxidation in PE placentas. Our study indicates that BER activity can be determined in placentas, and lower activity is present in PE compared with healthy. These findings should be followed up in prospective clinical investigations to examine BER’s role in the advancement of PE.

## 1. Introduction

DNA repair mechanisms are crucial for maintaining the integrity of DNA that is constantly under attack by various forms of DNA-damaging agents, including reactive oxygen species (ROS). Oxidative stress occurs when there is an imbalance between the production of ROS and the ability of the organism’s antioxidant capacity to neutralize them [1]. Prolonged exposure to ROS can lead to DNA damage, which, if left unrepaired, may result in the accumulation of mutations and the progression of various diseases like cancer [2]. One promising tool for studying DNA repair is the comet-based in vitro DNA repair assay [3,4]. This assay assesses the capacity of a cell or tissue extract to perform the initial steps of base excision repair (BER) or nucleotide excision repair (NER). More specifically, it assesses the damage recognition and incision activity of a protein extract on agarose gel-embedded substrate nucleoids (protein-depleted nuclei of supercoiled DNA) containing specifically induced DNA lesions (resulting from oxidation to study BER or UVC radiation or benzo[a]pyrene-diol epoxide treatment to study NER) [4].

Studies that used the comet-based in vitro DNA repair assay in exploring DNA repair activity in humans primarily focused on peripheral blood mononuclear cells (PBMCs) [5,6,7], but there are also examples in which solid tissues, such as colon biopsy samples, were analyzed [8,9]. The ability of the assay to study solid tissues was shown only recently. We were interested in optimizing the protocol for human placental tissue because it is considered a non-invasive tissue for human biomonitoring early-life exposures and studying molecular mechanisms underlying prenatal disorders [10]. Therefore, the aim of our study was to further optimize the comet-based in vitro DNA repair assay for placental tissues.

In this study, we focused on the assessment of BER activity and applied the newly modified assay to placentas affected by pre-eclampsia (PE) in comparison to placentas from normal pregnancies to additionally show its value in clinical applications. PE is a multifactorial disease affecting 1–10% of pregnancies and responsible for about 500,000 and 70,000 fetal and maternal deaths, respectively, globally each year [11,12]. The precise pathogenesis of the disease has not been entirely elucidated yet, but an abnormal placental development (also known as placentation) during the first trimester has been considered the leading cause of PE [12]. As a result of impaired placentation, abnormal placental perfusion will result in hypoxia and re-oxygenation, leading to the generation of oxidative stress [13]; indeed, it has previously been reported that PE is accompanied by higher levels of oxidative DNA damage [14,15]. Additionally, it has been suggested that the detection of (oxidation) DNA damage in early pregnancy may be useful in predicting PE [16]. The predominant pathway to repair oxidative stress-induced DNA damage (including 8-oxo-7,8-dihydro-2′-deoxyguanosine, 8-oxodG) in mammalian cells is BER, which suggests that BER may play a role in the etiology of PE.

In the first instance, we describe the various optimization steps that were undertaken, including the optimization of the protein concentration of the placental extract and the use of aphidicolin (APC) to potentially increase sensitivity [17]. Moreover, we conducted expression analyses of the genes involved in the early stages of BER (i.e., *OGG1*, *APEX1*, *NEIL1*, *PARP1,* and *MUTYH*) and assessed various oxidative stress markers to demonstrate that our new DNA repair marker correlates well with other biomarkers and has an added value in biomarker analysis.

## 2. Results

### 2.1. Optimization of the BER Assay for Placental Tissues

**Figure 1:**
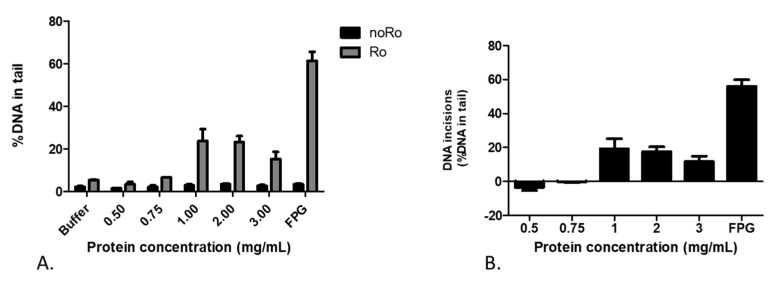
(**A**) Exposed (Ro) and non-exposed (noRo) cells in response to different concentrations of placental extract (mg/mL). Results are expressed as %DNA in tail and presented as the mean + SEM from two independent experiments. (**B**) DNA incision activity of different placental extracts (mg/mL) presented the mean value + SEM of two independent experiments.

Initially, a series of placental extract concentrations were tested in order to determine the most optimal protein concentration, e.g., the concentration that distinguishes best between the incision activity on exposed and non-exposed substrate cells, thus detecting the highest net DNA repair incision activity (Figure 1A).

The incision activities detected for the extracts of 1 mg/mL and 2 mg/mL of proteins (see Figure S1E,F for pictures of comets) were very similar (Figure 1B). For the representative pictures of comet tails obtained after incubation with Fpg or buffer, please refer to Figure S1A–D.

**Figure 2:**
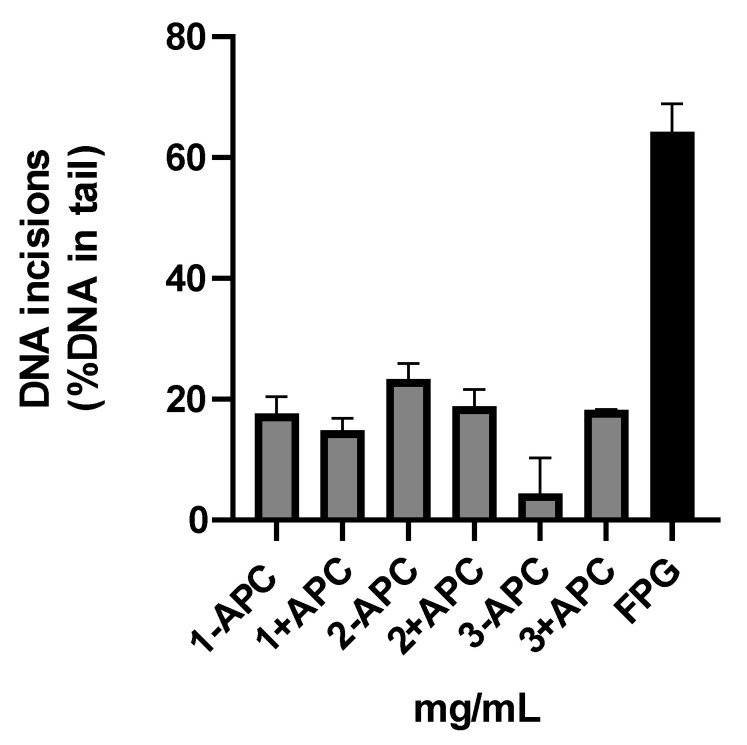
DNA incision activity of placental extracts (3, 2, and 1 mg/mL) incubated with (+APC) and without (−APC) 1.5 µM aphidicolin (APC) in DMSO. Results are presented as the mean value + SEM of two independent experiments.

Additionally, we tested whether non-specific enzyme activity was present in the extracts. We used APC in DMSO (final concentration 1.5 µM) as previously described [17], but no difference was observed compared to the samples that were not treated with APC (Figure 2). Slightly higher DNA incision activity was detected when using the 2 mg/mL protein concentration.

**Figure 3:**
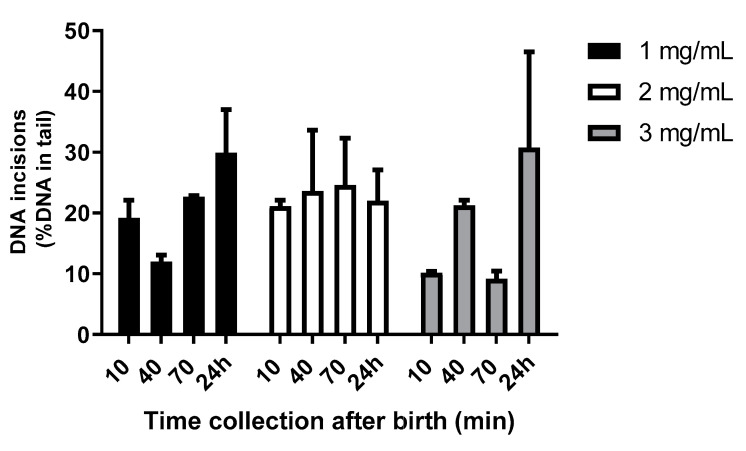
DNA incision activity dependent on time collection (10, 40, 70 min, and 24 h) after birth at three different placental extract concentrations (1, 2, and 3 mg/mL). The results are presented as the mean value + SEM of two independent experiments.

Finally, the effect of the time collection of the placental tissue on the BER activity was tested, with samples collected from the placentas at 10 min, 40 min, 70 min, and 24 h after birth. Although there were no statistically significant differences for each concentration over time, the detected DNA incision activity for 2 mg/mL was more consistent over time as compared to 1 and 3 mg/mL (Figure 3). For further analysis, 2 mg/mL was used as a protein concentration of the placental tissue extracts.

### 2.2. Applying the Assay to a Case Study on Pre-Eclampsia

#### 2.2.1. BER Incision Activity

**Figure 4:**
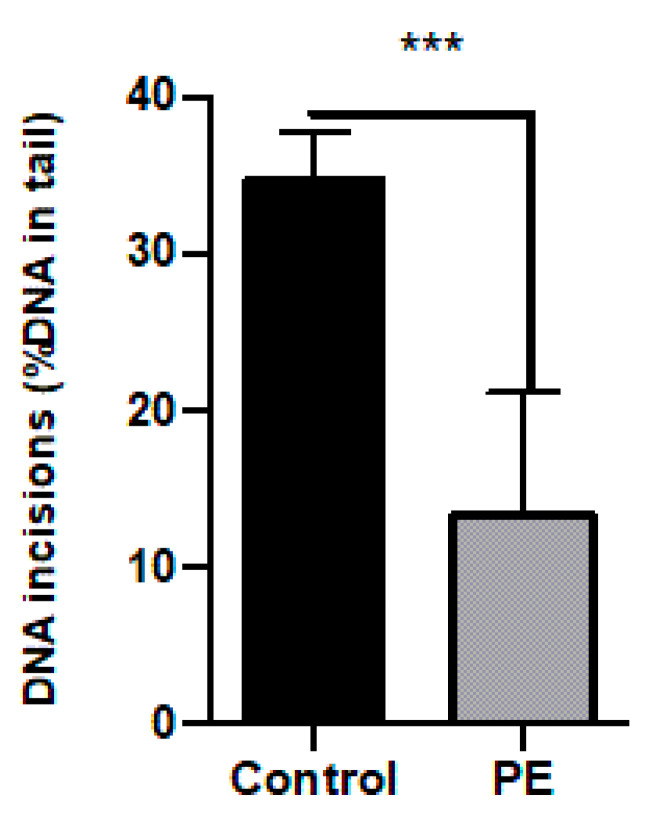
The BER incision activity in control placentas (*n* = 8) and placentas affected by pre-eclampsia (PE) placenta (*n* = 7) detected using the comet-based in vitro DNA repair assay. The results are presented as the mean value per group, and the bars represent the standard error. *** *p* ≤ 0.001.

BER activity of PE-complicated placentas was significantly lower compared to controls (*p* < 0.001). The detected BER incision activity in PE was only 32.5% of the BER incision activity detected in the control samples (Figure 4) (representative comet tails are shown in Figure S2).

#### 2.2.2. BER-Related Gene Expression

**Figure 5:**
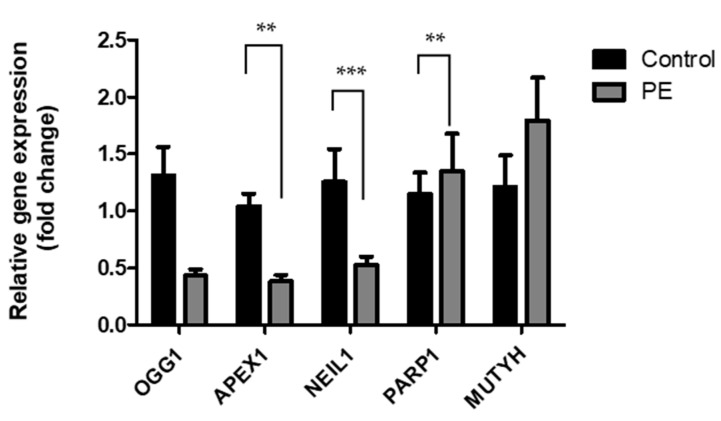
The relative expression levels of BER-related genes in healthy (*n* = 11) and pre-eclamptic (PE; *n* = 9) pregnancies. The target genes of interest were compared to the housekeeping gene β-actin. All samples were analyzed in triplicate. ** *p* ≤ 0.01, *** *p* ≤ 0.001.

In line with the lower DNA repair activity in the PE placenta, the gene expression levels of two of the primary DNA glycosylases, *OGG1* and *NEIL1*, and the endonuclease *APEX1*, were significantly lower in PE (Figure 5). More specifically, the expression levels were about half of the expression found in healthy pregnancies (*p* = 0.007, *p* < 0.0001, and *p* = 0.03, respectively). In contrast, *PARP1* and *MUTYH* expressions were not statistically different.

**Figure 6:**
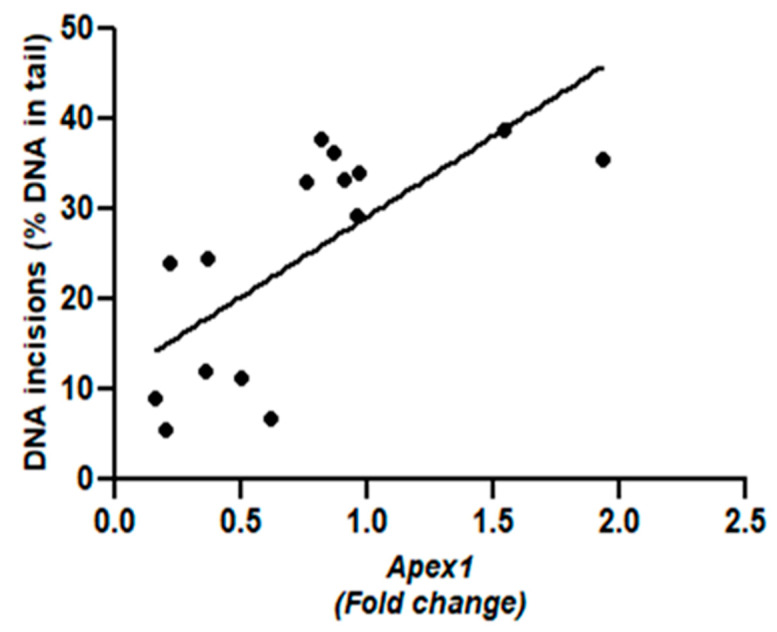
Positive correlation between the BER activity and the relative gene expression of *APEX1* (*r* = 0.72; *p* = 0.0027).

*APEX1* expression significantly correlated with the BER activity (Figure 6; *r* = 0.72, *p* = 0.0027). Although the expressions of *OGG1* and *NEIL1*, also showed a positive trend with BER activity, these relationships did not reach statistical significance.

#### 2.2.3. Markers of Oxidative Stress

**Figure 7:**
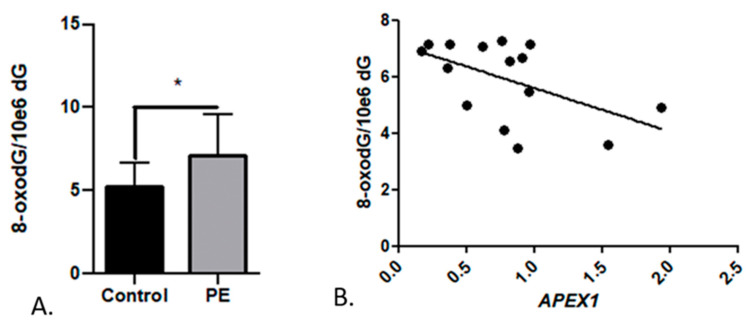
Average 8-oxodG levels (**A**) in healthy control placentas (*n* = 13) compared against pre-eclamptic placentas (PE; *n* = 10). Error bars represent the standard error; * *p* < 0.05. (**B**) Negative correlation between 8-oxodG levels and *APEX1* expression (*r* = −0.53; *p* = 0.043).

Pre-eclamptic placentas were shown to have 1.4-fold higher levels of 8-oxodG compared to healthy placentas (*p* = 0.037; Figure 7A), with an average of 7.0 ± 0.8 and 5.2 ± 0.4 8-oxodG per 10^6^ nucleotides, respectively. Additionally, a negative correlation was achieved between the 8-oxodG levels and *APEX1* relative gene expression (Figure 7B).

**Figure 8:**
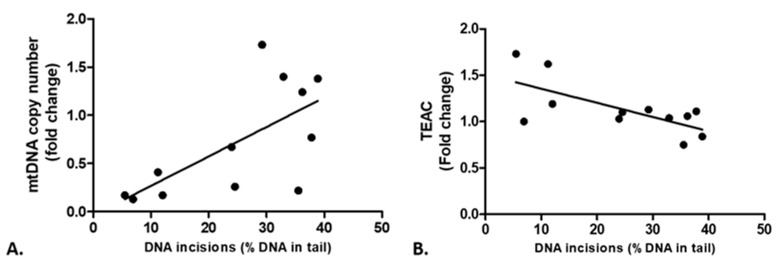
Correlations between the BER activity of the overall study group (healthy and preeclampsia combined) and (**A**) the mitochondrial DNA (mtDNA) copy number (fold change) (*r* = −0.67; *p* = 0.019), or (**B**) the Trolox equivalent antioxidant capacity (TEAC) (*r* = -0.68; *p* = 0.015).

In the main study [18], the mitochondrial DNA copy number of placentas complicated with PE was 3.5-fold lower compared to healthy placentas (average fold change was 0.29 ± 0.06 and 1.00 ± 0.16, respectively; *p* = 0.001). Interestingly, lower BER activity in PE patients was related to a lower mitochondrial copy number (Figure 8A; *r* = 0.67, *p* = 0.019).

Furthermore, in that same study [18], the total antioxidant capacity, assessed as TEAC (Trolox Equivalent Antioxidant Capacity), of the PE samples increased compared to the control samples; observing a fold change of 1.26 ± 0.10 and 1.00 ± 0.04 correspondingly (*p* = 0.02). Interestingly, BER activity and TEAC were inversely associated (*r* = −0.68, *p* = 0.015; Figure 8B).

## 3. Discussion

This is the first study to phenotypically assess BER incision activity in placental tissue, using the comet assay-based in vitro DNA repair assay. The placenta can be collected non-invasively and is a valuable tissue to assess early-life exposures as well as study underlying mechanisms of pregnancy-related disorders. For instance, the placenta is considered to play a main role in the development of PE. PE is characterized by ROS formation and oxidative stress, which leads to DNA lesions that are predominantly repaired by BER. To show the clinical value of the newly modified assay, BER activity was assessed in PE-complicated and healthy placentas. We indeed detected significantly less BER activity in pre-eclamptic placentas compared to controls, which was confirmed by significantly lower gene expression levels of *APEX1*, *OGG1*, and *NEIL1*. Additionally, the lower levels of DNA repair in PE were associated with increased levels of 8-oxodG, lower mitochondrial copy number, and a higher total antioxidant capacity in pre-eclamptic placentas.

### 3.1. Optimized Comet Assay-Based In Vitro DNA Repair Assay for Placental Tissues

The comet assay-based DNA repair assay is a valuable technique employed in molecular biology and genetic investigations for the evaluation of intracellular DNA repair mechanisms [4]. Since the principle of the assay is based on the ability of the protein extracts to recognize and incise the induced lesions in substrate DNA, the main optimization step was to determine the optimal protein concentration of the placental extracts. This optimization was crucial for the assay for several reasons: primarily to ensure high sensitivity and specificity in determining subtle changes in DNA repair efficiency and secondly to minimize background noise, where too high concentrations could lead to higher background noise due to non-specific enzyme activity and subsequently an inaccurate assessment of the DNA repair activity.

A range of concentrations between 0.5 and 3 mg/mL were tested based on reported protein concentrations for previously tested tissues [4]. It was observed that samples incubated with 2 mg/mL showed more reproducible data and consistency over time. None of the protein concentrations showed the presence of a significant amount of non-specific enzyme activity, which has previously been observed for other tissue types such as colon and lung tissue [19].

Previous studies have shown that the interval to sample collection after birth can be crucial depending on the molecular marker of interest. For instance, increased lipid peroxidation as a marker of oxidative stress was reported about 20 min after delivery. Since oxidative stress can trigger BER, this might explain why we see a trend of an increase in the BER activity with longer time intervals after birth, especially from 70 min onwards (Figure 3). There is no experimental evidence for a direct effect of delayed placental collection on gene expression levels, but translation and protein synthesis have been reported to be suppressed and thus remain stable within 30 min after delivery of the placenta [20]. Based on our findings and previous literature, we advise collecting placental samples within 30 min after birth.

### 3.2. The Clinical Value of the BER Assay for Placental Tissues

The second part of this study tested the newly optimized protocol in placental tissue of PE pregnancies. Lower BER activity was observed in PE placentas compared to controls (Figure 4), which was consistent with lower mRNA levels of *APEX1*, *OGG1*, and *NEIL1* (Figure 5). The role of both *OGG1* and *NEIL1*, as bifunctional glycosylases in BER, is to excise 8-oxoG lesions and promote β-elimination to produce a 3-deoxyribose phosphate (3-dRP) ends [21]. A decreased expression of *OGG1* in humans has indeed been linked to impaired 8-oxoG repair [22]. The downregulation of *NEIL1* was previously reported to be associated with increased oxidation DNA damage [23]. *APEX1* is the dominant AP endonuclease in human cells to incise the AP site after the removal of the damaged base after excision has taken place with a monofunctional DNA glycosylase [24].

In our study, a positive correlation between *APEX1* and BER activity was observed (Figure 6), indicating that *APEX1* can serve as a determinant for the BER levels detected in PE placentas. However, in regard to the genetic polymorphism of *APEX1* in PE, the findings are quite inconsistent, as there are studies that do not find a significant difference between PE and normotensive women [25,26,27]. PE has been widely characterized by maternal endothelial dysfunction in the placenta [28,29,30], and it has been previously suggested that the dysregulation of endothelial cells caused by hypoxia may be related to the downregulation of *APEX1* [31]. The gene expressions of both *OGG1* and *APEX1* have been previously examined in PE-deficient placentas at the maternal-fetal interface, and it was observed that they were overexpressed in decidual cells and cytotrophoblasts [32]. This is in contrast to the lower levels of gene expression observed in our current study, However, the authors suggested that *OGG1* and *APEX1* may play a role in the BER mechanism of the placenta to combat ROS-induced DNA damage [32]. The contradictive results may be related to differences in clinical characteristics, including the gestational age, maternal age, type of delivery, or sex of the baby.

As pre-eclamptic placentas are characterized by excessive oxidative stress [18], an overall lower BER activity can lead to DNA damage accumulation and eventually mutations [33]. In our study, PE-complicated placentas indeed showed higher 8-oxdG levels compared to control (Figure 7A), which is in line with oxidative stress-induced DNA damage as previously reported in PE patients [14,34,35,36]. Additionally, we observed an association between increased 8-oxodG levels and reduced *APEX1* expression (Figure 7B), which is in line with a previous study that also reports a downregulation of BER enzymes in the presence of increased DNA damage [22]. In our previous studies [18] and the current study, a lower mtDNA copy number (as an indicator of oxidative stress) was observed in the PE placentas compared to controls. As widely acknowledged by now, mitochondria also have the ability to repair their DNA by BER, perhaps with even higher capacity than nuclear DNA [37,38,39]. It is known that key components of BER are encoded by nuclear genes and translocated into the mitochondria [38]. If mtDNA is not efficiently repaired, mitophagy is suggested to take place [38]; therefore, the loss of mtDNA copy number that we observed in PE placentas could be due to an excessive amount of unrepaired oxidative mitochondrial DNA damage and/or lower BER activity in mitochondria. This hypothesis was unfortunately not validated in our study and deserves further attention. However, we did find a significant positive correlation between the mtDNA copy number and BER activity in whole-cell extracts for placental tissue (Figure 8A).

To prevent oxidation-induced DNA damage, oxidative stress is commonly neutralized by antioxidant activity [40,41]. While ROS and other oxidative species are important for the cellular signaling and regulation of gene expression in the placenta during pregnancy; at the same time, the placenta generates abundant antioxidants to prevent oxidative stress [42]. In a previous study, an increased total antioxidant capacity was observed in PE placentas [18]. This is in line with a study by Llurba et al., which presented elevated antioxidant activity in erythrocytes of PE patients [43]. These data indicate that the higher levels of oxidative stress in the PE placentas potentially correlate with the upregulation of antioxidant defenses.

Studies have reported that lowering oxidative stress by antioxidants enhances DNA repair activity [44,45]. Similarly, here, we detected a negative correlation between the antioxidant activity with the overall BER activity in the placentas (Figure 8B). This inverse relation suggests that when the level of antioxidants is sufficiently high to prevent oxidative stress, DNA repair may not be needed. However, when antioxidant defenses fail, and oxidative stress increases, this might trigger DNA repair. Indeed, previous studies showed that DNA repair could be induced relatively quickly by specific DNA-damaging triggers (as previously reviewed [46]).

### 3.3. Concluding Remarks

As mentioned before, the value of using placental tissues is the non-invasive collection of the biomatrix and the ability to study molecular markers early in life in relation to exposure or disease. Previous studies have assessed DNA repair activity in placental tissue in an indirect way via the study of gene expression [23,24,25] or protein expression [47], and genetic variants [24,26,27], However, studying gene variants or expression does not necessarily give any indication of enzyme activity. The lack of correspondence between DNA repair gene expression and enzyme activity has been discussed before [46]. The expression and activity of a particular protein in the cell depends not only on genetic variations [48]. Moreover, enzyme activity is not solely determined by the quantity of protein but can be influenced by post-transcriptional modifications and the presence of various co-factors. Although assessing gene expression can give valuable information, the measurement of BER activity, as performed in this study, allows us to capture the activity of the BER process and the multiple enzymes involved. It is worth mentioning that this study exclusively investigated BER via the comet-based in vitro DNA repair assay and not NER. The timing of tissue collection after delivery is of particular importance with respect to NER activity since the rapid decline in ATP levels within the first 10 min post-delivery was previously reported [20]. This temporal factor is significant due to the ATP-dependent nature of the initial steps of NER.

Although we only showed the value of the comet assay-based BER assay with placental tissues in a clinical setting, we are convinced that the effects of exposures during pregnancy (e.g., smoking, dietary habits, occupational exposures, etc.) on DNA repair can be assessed via this same optimized assay in the framework of human biomonitoring studies.

In conclusion, we are the first to optimize and use the comet assay-based in vitro DNA repair assay with placental tissue and show its clinical value. Our study showed considerably lower BER activity in PE-diseased placentas, which was associated with lower expressions of BER-related genes. Furthermore, we observed a significant negative relation between the total antioxidant activity and BER, indicating an interplay of these defense mechanisms to cope with the oxidative stress in the placenta. Correlations between BER (both enzyme activity and gene expression) and oxidative stress markers (oxidized DNA and mtDNA copy number) in the placenta suggest that BER plays an important role in the progression of PE and shows the value of including BER activity as an additional biomarker in future clinical studies.

## 4. Materials and Methods

### 4.1. Placental Specimen

**Figure 9:**
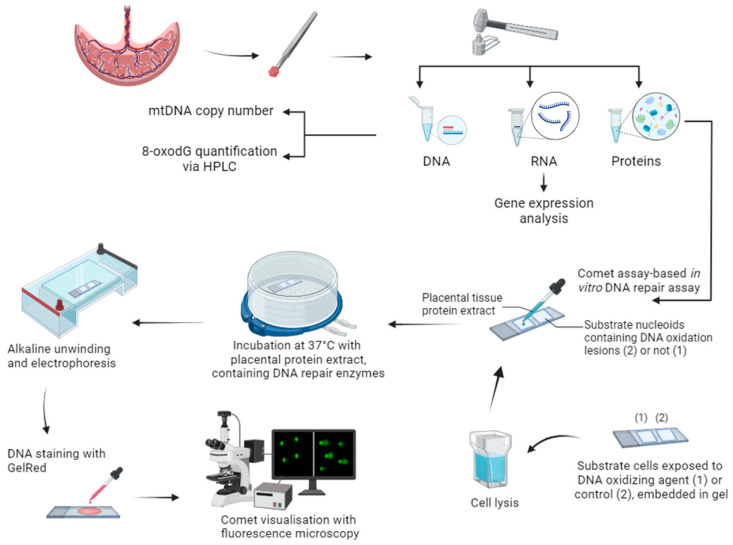
Visual illustration of the experimental setup and methodology. Placental biopsies underwent pulverization, to subsequently isolate DNA for mtDNA copy number and 8-oxodG quantification analysis. Furthermore, RNA extraction enabled gene expression analysis, in parallel to protein extraction for conducting the comet-based in vitro DNA repair assay on substrate cells containing DNA lesions induced by an oxidizing agent. This schematic overview was created in BioRender.com.

To optimize the comet assay-based in vitro DNA repair assay for the use of placental tissues, 4 disease-free placentas were obtained via the Maastricht University Medical Center+. Placental tissue measuring less than one square centimeter was cut from the paracentral zone of the maternal side of the placenta. Following collection, the samples underwent thorough washing in ice-cold phosphate-buffered saline (PBS), followed by removal of the basal plate. Subsequently, the samples were snap-frozen in liquid nitrogen. This snap-freezing procedure was executed at varying time intervals postpartum, specifically at 10, 40, 70 min, and 24 h after delivery. Snap-frozen tissues were pulverized while in a frozen state using a mortar and then preserved at −80 °C until further analysis (Figure 9).

For the case study, 11 healthy and 9 PE placentas were obtained from the Maastricht University Medical Center+. Placental samples (<1 cm^2^) were obtained (within a 15 min timeframe) postpartum from the paracentral region of the placenta on the maternal side, ground under liquid nitrogen, and stored at −80 °C as previously described [18]. Pre-eclampsia (PE) was identified following the guidelines of the International Society for the Study of Hypertension in Pregnancy (ISSHP) criteria, as previously described [18]. This study and our analyses received approval from the Medical Ethics Committee of Maastricht University Medical Center+ (Maastricht, The Netherlands, No. 16-4-047), and participants provided oral informed consent before placenta inclusion. Clinical characteristics of the study subjects are summarized in Table 1.

### 4.2. DNA Repair Activity/BER Incision Activity

#### 4.2.1. Principle of the Assay

The principle of the comet-based in vitro DNA repair assay is that protein extracts from tissues or cells are incubated with agarose-embedded substrate cells that contain specific DNA lesions (in this study, DNA oxidation lesions, mainly in the form of 8-oxoguanine (8-oxoG)). The DNA repair proteins recognize the DNA damage and will subsequently make DNA excisions and incisions, which are measured by the comet assay as strand breaks after electrophoresis [4].

#### 4.2.2. Substrate Cells Preparation

The preparation of the substrate cells was performed as previously described [4]. Briefly, adenocarcinoma human alveolar basal epithelial A549 cells were exposed to either photosensitizer 1 μM Ro 19-8022 (Hoffmann La Roche, Basel, Switzerland) or PBS (representing the negative control). After washing, the cells were exposed to light from a 500 W tungsten halogen lamp for 5 min on ice at a distance of 33 cm. Next, cells were washed, harvested through trypsinization, and counted. The cells were diluted to a concentration of 2 × 10^5^ cells/mL, using freezing medium (50% RPMI, 40% FBS, 10% DMSO) (purchased from Gibco via Fisher Scientific, Landsmeer, The Netherlands; Sigma-Aldrich, Zwijndrecht, The Netherlands; Merck, Darmstadt, Germany). The resulting substrate cells were slowly frozen with the freezing container Mr. Frosty (Thermo Fisher Scientific, Zwijndrecht, The Netherlands) and stored at −80 °C until further use.

#### 4.2.3. Tissue Extract Preparation

About 30 mg of the ground tissue was treated with 100 μL extraction buffer A (45 mM HEPES, 0.4 M KCl, 1 mM EDTA-Na_2_∙2H_2_O, 0.1 mM DTT, 10% (*v*/*v*) glycerol (Sigma-Aldrich, Zwijndrecht, The Netherlands)), vortexed, and snap-frozen. After thawing, 30 μL of buffer A containing 1% Triton X-100 was added and incubated for 10 min on ice. The tissue extracts were collected after centrifugation at 15,000× *g* for 5 min at 4 °C. The protein concentration of the extract was determined at 750 nm by a Lowry-based DC Protein Assay Kit (Bio-Rad, Lunteren, The Netherlands) against a bovine serum albumin (BSA; Sigma-Aldrich, Zwijndrecht The Netherlands) standard curve.

#### 4.2.4. Extract Incubation

Substrate cells were thawed and washed with cold (4 °C) PBS. The cells were resuspended in 300 μL cold PBS and mixed with 700 μL of 1% low melting point (LMP) agarose (37 °C). Subsequently, 70 μL of Ro 19-8002 exposed (Ro) and non-exposed (noRo) cells were pipetted on 76 × 26 mm glass slides (Thermo Fisher Scientific, Zwijndrecht, The Netherlands) and covered with 24 × 24 mm coverslips. The slides were put on an ice-cooled plate for 5–10 min to solidify and then subjected to lysis (2.5 M NaCl, 0.1 M EDTA-Na_2_, 19 mM Trizma base and 1% Triton X-100 (Sigma-Aldrich, Zwijndrecht, The Netherlands)) for 1 h at 4 °C. Subsequently, the slides were washed with 1× Buffer B (40 mM HEPES, 0.5 mM EDTA-Na_2_, 0.2 mg/mL BSA, 0.1 M KCl) twice for 5 min at 4 °C.

The protein concentration of the tissue extracts was adjusted to the desired concentrations (i.e., 0.5–3 mg/mL to test the optimal protein dilution and to 2 mg/mL for further analysis) with buffer A containing 0.25% Triton X-100 and subsequently 4× diluted with buffer B. Each gel was incubated with 50 μL tissue extract, formamidopyrimidine DNA glycosylase (Fpg; as incubation reaction control when incubated with Ro-cells), or buffer A containing 0.25% Triton X-100 (as background control when incubated with noRo-cells or treatment control when incubated with Ro cells) at 37 °C for 30 min. Next, the samples were placed on ice to stop the enzymatic reaction and incubated in electrophoresis solution (0.3 M NaOH, 1 mM EDTA-Na_2_) for 40 min at 4 °C. Subsequently, the gels were subjected to electrophoresis at 1 V/cm for 30 min at 4 °C. The gels were neutralized in cold (4 °C) 1× PBS and washed in cold dH_2_O, each for 10 min.

#### 4.2.5. Staining and Comet Analysis

Each gel was stained with GelRed (Sigma-Aldrich, Zwijndrecht, The Netherlands) (1:3333 in water) for 20 min before visualization under a fluorescence microscope, and 50 comets/gel were scored using Comet Assay III software (INSTEM, Staffordshire, UK).

For the calculation of the final DNA repair incision activity, firstly, the tail intensity (TI) median was calculated from 50 comets/gel, and the mean of the two replicates was considered. Next, the background level (TI_noRo/Buffer_) was subtracted from all data, after which the following formula was used to determine the final BER incision activity:

$$\mathrm{BER}\mathrm{incision}\mathrm{activity}=({TI}_{Ro/Extract}-{TI}_{noRo/Extract})-{TI}_{Ro/Buffer}$$

where the nucleoids containing 8-oxoG lesions incubated with the extracts are represented by TI_Ro/Extract_, TI_noRo/Extract_ refers to non-exposed nucleoids incubated with extract, and TI_Ro/Buffer_ represents Ro 198002-exposed nucleoids incubated with buffer. DNA repair incision activity was normalized as previously described [4]. In short, the activity detected for the incubation reaction control Fpg per assay (ranging from 64 to 79%) was used to perform the normalization. Values for the background control and treatment control ranged from 1.9 to 3.35% and from 4.9 to 8.65%, respectively, before Fpg correction.

### 4.3. Gene Expression Analysis of DNA Glycosylases

RNA was isolated and diluted to a final concentration of 100 ng/μL as previously described [18]. Next, 500 ng of RNA was converted to cDNA with an iScriptTM cDNA synthesis kit (Bio-Rad, Lunteren, The Netherlands), using the following thermal cycle conditions: 5 min at 25 °C (priming), 20 min at 46 °C (reverse transcription) and finally 1 min at 95 °C (RT inactivation). The resulting cDNA samples were diluted 1:50 and stored at −20 °C until further processing expression was determined using a LightCycler480 (Hoffmann La Roche, Basel, Switzerland). Then, 11 ng of cDNA was amplified using a PCR reaction volume of 10 μL with a final concentration of 300 nM forward and reversed gene-specific primers (Eurofins Genomics, Ebersberg) (Refer to Table 2 for sequences) and 2× SensimixTM Syber & Fluorescein mix (Bioline, Alphen aan de Rijn, The Netherlands). The PCR thermal cycles included: 10 s at 95 °C, 45 cycles of 10 s at 95 °C, and 20 s at 60 °C. Samples were analyzed in triplicate. Ct values were achieved using the LC48Conversion program and the efficiency of the PCR reaction was evaluated by LinRegPCR. The expression level of the targeted genes was normalized against the housekeeping gene *beta-actin*. The relative fold expression was determined with the delta-delta Ct method (2^−ΔΔCt^).

### 4.4. Determination of Oxidative Stress Parameters

#### 4.4.1. 8-oxodG Lesion Quantification

Genomic DNA was isolated from frozen ground placenta tissues (~30–80 mg) using standard extraction procedures [49]. To minimize artificial induction of 8-oxodG, radical-free phenol, 1 mM deferoxamine mesylate, and 20 mM TEMPO (2,2,6,6-tetramethylpiperidine-N-oxyl; Aldrich, Steinheim, Germany) were used during the DNA extraction, in correspondence with the recommendations made by the European Standards Committee on Oxidative DNA Damage (ESCODD [50]). HPLC with electrochemical detection (ECD) was performed as described earlier [51] to detect the base oxidation product 8-oxodG. Normal nucleotides were detected by UV absorbance. The amount of DNA damage was expressed as the number of 8-oxodG per 10^6^ unmodified dG.

#### 4.4.2. Determination of Mitochondrial DNA (mtDNA) Copy Number and the Trolox Equivalent Antioxidant Capacity (TEAC)

These measurements were conducted as described in a previous main study [18] from which we analyzed a sub-group. DNA was used for quantitative PCR amplification with mtDNA cytochrome C oxidase subunit 2 (COXII) and genomic DNA (gDNA) RPL13A specific primer. The mtDNA/gDNA ratio was achieved with the division of the relative quantity of mtDNA by the relative quantity of gDNA. The antioxidant capacity was determined as described in a previous main study [18] from which we analyzed a sub-group.

### 4.5. Statistical Analysis

The results are presented as the mean ± standard error (SE). Differences between healthy and PE placentas were assessed using t-test. Pearson correlation test was performed to study relationships between the various markers. A *p*-value < 0.05 was considered statistically significant, using SPSS Statistical software (version 27). The graphs were plotted with GraphPad Prism 5.

## Figures and Tables

**Table 1:** Study subjects’ clinical characteristics presented as the mean value ± standard deviation.

	Control (N = 11)	PE (N = 9)
Maternal age (years)	29 ± 4	30 ± 4
Maternal BMI (kg/m^2^)	25 ± 6	24 ± 4
GA (weeks)	39 ± 1	33 ± 4 **
Birth weight (kg)	3.3 ± 0.5	2.4 ± 1 *
CS (%)	55	44
IUGR (%)	0	50 *

Body mass index (BMI), gestational age (GA), cesarean section (CS), intrauterine growth restriction (IUGR). * *p* < 0.05; ** *p* < 0.01.

**Table 2:** Specific primers for qRT-PCR analysis. Final concentration of each primer is 300 nM.

Gene	Forward (5′→3′)	Reverse (5′→3′)
*OGG1*	TGGGGCATCGTACTCTAGC	AGATTGTCCAGAAGGCAGAAC
*APE1*	TGCCTTCAAGAGACCAAATGTT	CGCCACTGTACCCTTCCTT
*NEIL1*	GACTGGCGCTTTCTGATTTC	AGCCAAGCAACAACAACAAC
*PARP1*	CCACACACAATGCGTATGACT	CCACAGCAATCTTCGGTTATGA
*MUTYH*	AATTTCTTTCGGTCTCACATCTC	AAATAAGCACTTTACTAACAACAGGA
*B-ACTIN*	CACCCAAGAACAGGGTTTGT	TGGCCATGGGTATGTTGTTAA

## Data Availability

Data is contained within the article and supplementary material.

## Supplementary Materials

The supporting information can be downloaded at: https://www.mdpi.com/article/10.3390/ijms25010187/s1.

## References

[1] Birben E., Sahiner U.M., Sackesen C., Erzurum S., Kalayci O. (2012). Oxidative stress and antioxidant defense. World Allergy Organ. J..

[2] Liou G.Y., Storz P. (2010). Reactive oxygen species in cancer. Free Radic. Res..

[3] Collins A.R. (2004). The comet assay for DNA damage and repair: Principles, applications, and limitations. Mol. Biotechnol..

[4] Vodenkova S., Azqueta A., Collins A., Dusinska M., Gaivão I., Møller P., Opattova A., Vodicka P., Godschalk R.W.L., Langie S.A.S. (2020). An optimized comet-based in vitro DNA repair assay to assess base and nucleotide excision repair activity. Nat. Protoc..

[5] Fikrova P., Stetina R., Hrnciarik M., Hrnciarikova D., Hronek M., Zadak Z. (2013). DNA crosslinks, DNA damage and repair in peripheral blood lymphocytes of non-small cell lung cancer patients treated with platinum derivatives. Oncol. Rep..

[6] Slyskova J., Naccarati A., Pardini B., Polakova V., Vodickova L., Smerhovsky Z., Levy M., Lipska L., Liska V., Vodicka P. (2012). Differences in nucleotide excision repair capacity between newly diagnosed colorectal cancer patients and healthy controls. Mutagenesis.

[7] Stoyanova E., Pastor S., Coll E., Azqueta A., Collins A.R., Marcos R. (2013). Base excision repair capacity in chronic renal failure patients undergoing hemodialysis treatment. Cell Biochem. Funct..

[8] Slyskova J., Korenkova V., Collins A.R., Prochazka P., Vodickova L., Svec J., Lipska L., Levy M. (2012). Functional, genetic, and epigenetic aspects of base and nucleotide excision repair in colorectal carcinomas. Clin. Cancer Res..

[9] Slyskova J., Langie S.A.S., Collins A.R., Vodicka P. (2014). Functional evaluation of DNA repair in human biopsies and their relation to other cellular biomarkers. Front. Genet..

[10] Manokhina I., Del Gobbo G.F., Konwar C., Wilson S.L., Robinson W.P. (2017). Review: Placental biomarkers for assessing fetal health. Hum. Mol. Genet..

[11] Portelli M., Baron B. (2018). Clinical Presentation of Preeclampsia and the Diagnostic Value of Proteins and Their Methylation Products as Biomarkers in Pregnant Women with Preeclampsia and Their Newborns. J. Pregnancy.

[12] Rana S., Lemoine E., Granger J.P., Karumanchi S.A. (2019). Preeclampsia: Pathophysiology, Challenges, and Perspectives. Circ. Res..

[13] Cheng M.-H., Wang P.-H. (2009). Placentation abnormalities in the pathophysiology of preeclampsia. Expert Rev. Mol. Diagn..

[14] Chiarello D.I., Abad C., Rojas D., Toledo F., Vázquez C.M., Mate A., Sobrevia L., Marín R. (2018). Oxidative stress: Normal pregnancy versus preeclampsia. Biochim. Biophys. Acta Mol. Basis Dis..

[15] Kimura C., Watanabe K., Iwasaki A., Mori T., Matsushita H., Shinohara K., Wakatsuki A. (2012). The severity of hypoxic changes and oxidative DNA damage in the placenta of early-onset preeclamptic women and fetal growth restriction. J. Matern. Neonatal Med..

[16] Furness D.L.F., Dekker G.A., Hague W.M., Khong T.Y., Fenech M.F. (2010). Increased lymphocyte micronucleus frequency in early pregnancy is associated prospectively with pre-eclampsia and/or intrauterine growth restriction. Mutagenesis.

[17] Langie S.A.S., Cameron K.M., Waldron K.J., Fletcher K.P.R., von Zglinicki T., Mathers J.C. (2011). Measuring DNA repair incision activity of mouse tissue extracts towards singlet oxygen-induced DNA damage: A comet-based in vitro repair assay. Mutagenesis.

[18] Vangrieken P., Al-Nasiry S., Bast A., Leermakers P.A., Tulen C.B.M., Schiffers P.M.H., van Schooten F.J., Remels A.H.V. (2021). Placental Mitochondrial Abnormalities in Preeclampsia. Reprod. Sci..

[19] Gorniak J.P., Cameron K.M., Waldron K.J., von Zglinicki T., Mathers J.C., Langie S.A.S. (2013). Tissue differences in BER-related incision activity and non-specific nuclease activity as measured by the comet assay. Mutagenesis.

[20] Burton G., Sebire N., Myatt L., Tannetta D., Wang Y.-L., Sadovsky Y., Staff A., Redman C. (2014). Optimising sample collection for placental research. Placenta.

[21] Hao W., Wang J., Zhang Y., Wang C., Xia L., Zhang W., Zafar M., Kang J.Y., Wang R., Bohio A.A. (2020). Enzymatically inactive OGG1 binds to DNA and steers base excision repair toward gene transcription. FASEB J..

[22] Shah A., Gray K., Figg N., Finigan A., Starks L., Bennett M. (2018). Defective Base Excision Repair of Oxidative DNA Damage in Vascular Smooth Muscle Cells Promotes Atherosclerosis. Circulation.

[23] Maiti A.K., Boldogh I., Spratt H., Mitra S., Hazra T.K. (2008). Mutator phenotype of mammalian cells due to deficiency of NEIL1 DNA glycosylase, an oxidized base-specific repair enzyme. DNA Repair.

[24] Kladova O.A., Alekseeva I.V., Saparbaev M., Fedorova O.S., Kuznetsov N.A. (2020). Modulation of the Apurinic/Apyrimidinic Endonuclease Activity of Human APE1 and of Its Natural Polymorphic Variants by Base Excision Repair Proteins. Int. J. Mol. Sci..

[25] Michita R.T., Kaminski V.d.L., Chies J.A.B. (2018). Genetic Variants in Preeclampsia: Lessons from Studies in Latin-American Populations. Front. Physiol..

[26] Sandoval-Carrillo A., Méndez-Hernández E.M., Vazquez-Alaniz F., Aguilar-Durán M., Téllez-Valencia A., Barraza-Salas M., Castellanos-Juárez F.X., La Llave-León O., Salas-Pacheco J.M. (2014). Polymorphisms in DNA repair genes (APEX1, XPD, XRCC1 and XRCC3) and risk of preeclampsia in a Mexican mestizo population. Int. J. Mol. Sci..

[27] Vural P., Değirmencioğlu S., Doğru-Abbasoğlu S., Saral N.Y., Akgül C., Uysal M. (2009). Genetic polymorphisms in DNA repair gene APE1, XRCC1 and XPD and the risk of pre-eclampsia. Eur. J. Obstet. Gynecol. Reprod. Biol..

[28] Chambers J.C., Fusi L., Malik I.S., Haskard D.O., De Swiet M., Kooner J.S. (2001). Association of maternal endothelial dysfunction with preeclampsia. JAMA.

[29] Sanchez-Aranguren L.C., Prada C.E., Riãno-Medina C.E., Lopez M. (2014). Endothelial dysfunction and preeclampsia: Role of oxidative stress. Front. Physiol..

[30] Lamarca B. (2012). Endothelial dysfunction; an important mediator in the pathophysiology of hypertension during pre-eclampsia. Minerva Ginecol..

[31] Bhakat K.K., Mantha A.K., Mitra S. (2009). Transcriptional regulatory functions of mammalian AP-endonuclease (APE1/Ref-1), an essential multifunctional protein. Antioxid. Redox Signal..

[32] Tadesse S., Norwitz N.G., Guller S., Arcuri F., Toti P., Norwitz E.R., Kidane D. (2017). Dynamics of Base Excision Repair at the Maternal-Fetal Interface in Pregnancies Complicated by Preeclampsia. Reprod. Sci..

[33] Clancy S. (2008). DNA damage & repair: Mechanisms for maintaining DNA integrity. Nat. Educ..

[34] Hilali N., Kocyigit A., Demir M., Camuzcuoglu A., Incebiyik A., Camuzcuoglu H., Vural M., Taskin A. (2013). DNA damage and oxidative stress in patients with mild preeclampsia and offspring. Eur. J. Obstet. Gynecol. Reprod. Biol..

[35] Shehu C., Ekele B., Suleman B., Eze P., Burodo A., Suleiman B. (2020). A Comparative Study of Oxidative Stress in Preeclampsia and Normal Pregnancy. Sch. Int. J. Obstet. Gynecol..

[36] Tadesse S., Kidane D., Guller S., Luo T., Norwitz N.G., Arcuri F., Toti P., Norwitz E.R. (2014). In vivo and in vitro evidence for placental DNA damage in preeclampsia. PLoS ONE.

[37] de Souza-Pinto N.C., Hogue B.A., Bohr V.A. (2001). DNA repair and aging in mouse liver: 8-oxodG glycosylase activity increase in mitochondrial but not in nuclear extracts. Free Radic. Biol. Med..

[38] Rong Z., Tu P., Xu P., Sun Y., Yu F., Tu N., Guo L., Yang Y. (2021). The Mitochondrial Response to DNA Damage. Front. Cell Dev. Biol..

[39] Zhao L. (2019). Mitochondrial DNA degradation: A quality control measure for mitochondrial genome maintenance and stress response. Enzyme.

[40] Collins A.R. (1999). Oxidative DNA damage, antioxidants, and cancer. Bioessays.

[41] Sagun K.C., Cárcamo J.M., Golde D.W. (2006). Antioxidants prevent oxidative DNA damage and cellular transformation elicited by the over-expression of c-MYC. Mutat. Res. Mol. Mech. Mutagen..

[42] Wu F., Tian F., Lin Y., Xu W. (2015). Oxidative Stress: Placenta Function and Dysfunction. Am. J. Reprod. Immunol..

[43] Llurba E., Gratacós E., Martín-Gallán P., Cabero L., Dominguez C. (2004). A comprehensive study of oxidative stress and antioxidant status in preeclampsia and normal pregnancy. Free Radic. Biol. Med..

[44] Kaur P., Purewal S.S., Sandhu K.S., Kaur M. (2019). DNA damage protection: An excellent application of bioactive compounds. Bioresour. Bioprocess..

[45] Riklis E., Emerit I., Setlow R. (1996). New approaches to biochemical radioprotection: Antioxidants and DNA repair enhancement. Adv. Space Res..

[46] Collins A.R., Azqueta A., Langie S.A.S. (2012). Effects of micronutrients on DNA repair. Eur. J. Nutr..

[47] Fujimaki A., Watanabe K., Mori T., Kimura C., Shinohara K., Wakatsuki A. (2011). Placental oxidative DNA damage and its repair in preeclamptic women with fetal growth restriction. Placenta.

[48] Capp J. (2021). Interplay between genetic, epigenetic, and gene expression variability: Considering complexity in evolvability. Evol. Appl..

[49] Hoff-Olsen P., Mevåg B., Staalstrøm E., Hovde B., Egeland T., Olaisen B. (1999). Extraction of DNA from decomposed human tissue: An evaluation of five extraction methods for short tandem repeat typing. Forensic. Sci. Int..

[50] Lunec J. (1998). ESCODD: European Standards Committee on Oxidative DNA Damage. Free Radic. Res..

[51] de Kok T.M., ten Vaarwerk F., Zwingman I., van Maanen J., Kleinjans J. (1994). Peroxidation of linoleic, arachidonic and oleic acid in relation to the induction of oxidative DNA damage and cytogenetic effects. Carcinogenesis.

