# Supplementary material for: Optimizing the Comet Assay-Based In Vitro DNA Repair Assay for Placental Tissue: A Pilot Study with Pre-Eclamptic Patients
Source: Int J Mol Sci. 2023 Dec 22;25(1):187. doi: 10.3390/ijms25010187 (PMC10779140; doi:10.3390/ijms25010187)
Supplement: Supplementary file 1 [file ijms-25-00187-s001.zip › ijms-2744069-supplementary.pdf]

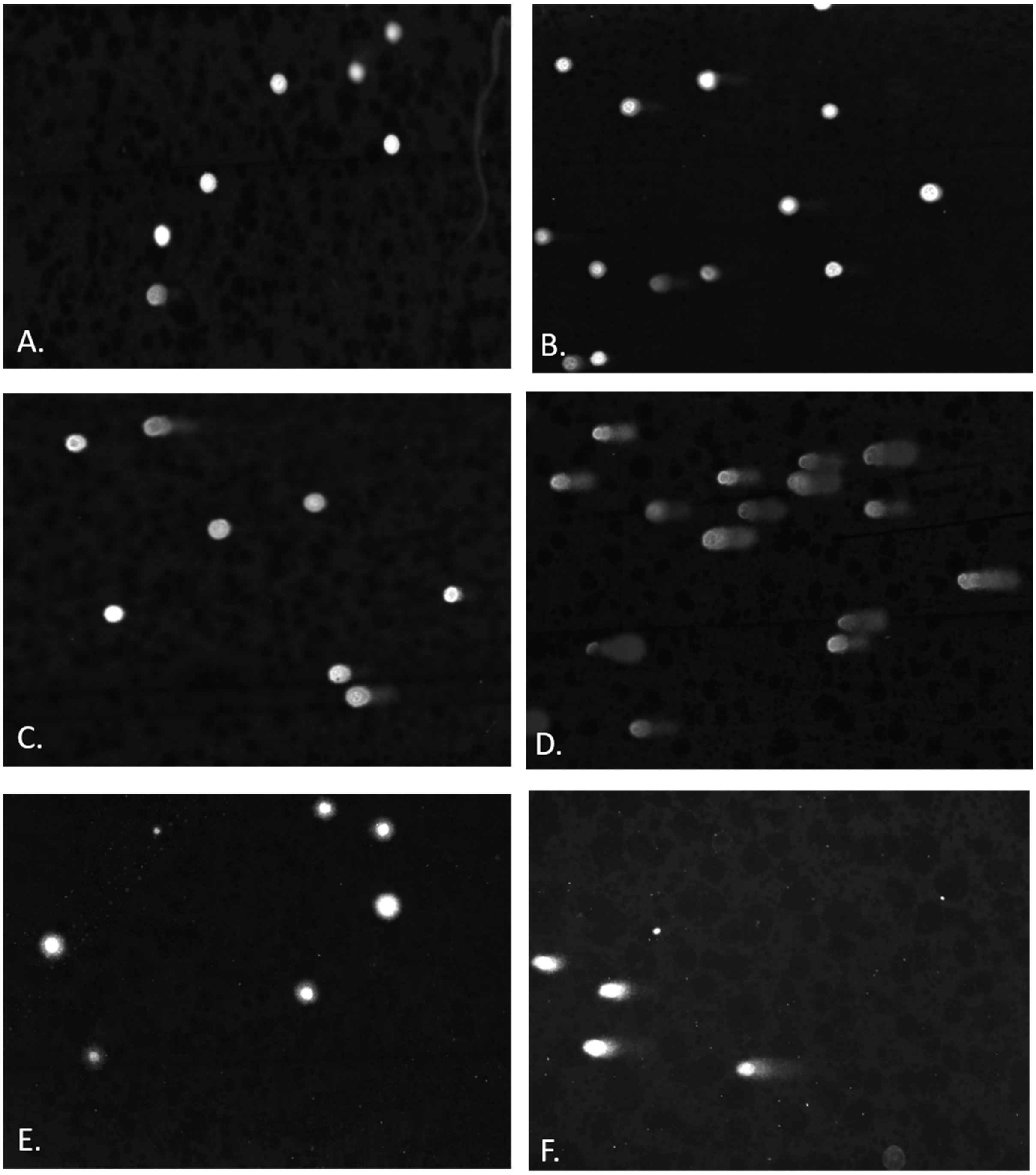

Figure S1. Representative pictures of A549 cells used as substrates in the DNA repair experiment, incubated with control solutions (buffer and FPG) or placental tissue extract at 2 mg/mL. A) Non exposed (noRo) cells incubated with buffer (negative control) ; B) Exposed (Ro) cells incubated with buffer; C) Non exposed (noRo) cells incubated with Fpg (positive control); D) Exposed (Ro) cells incubated with Fpg. E) Non-exposed cells (noRo) incubated with placental tissue extract at 2 mg/mL; F) Exposed (Ro) cells incubated with placental tissue extract at 2 mg/mL.

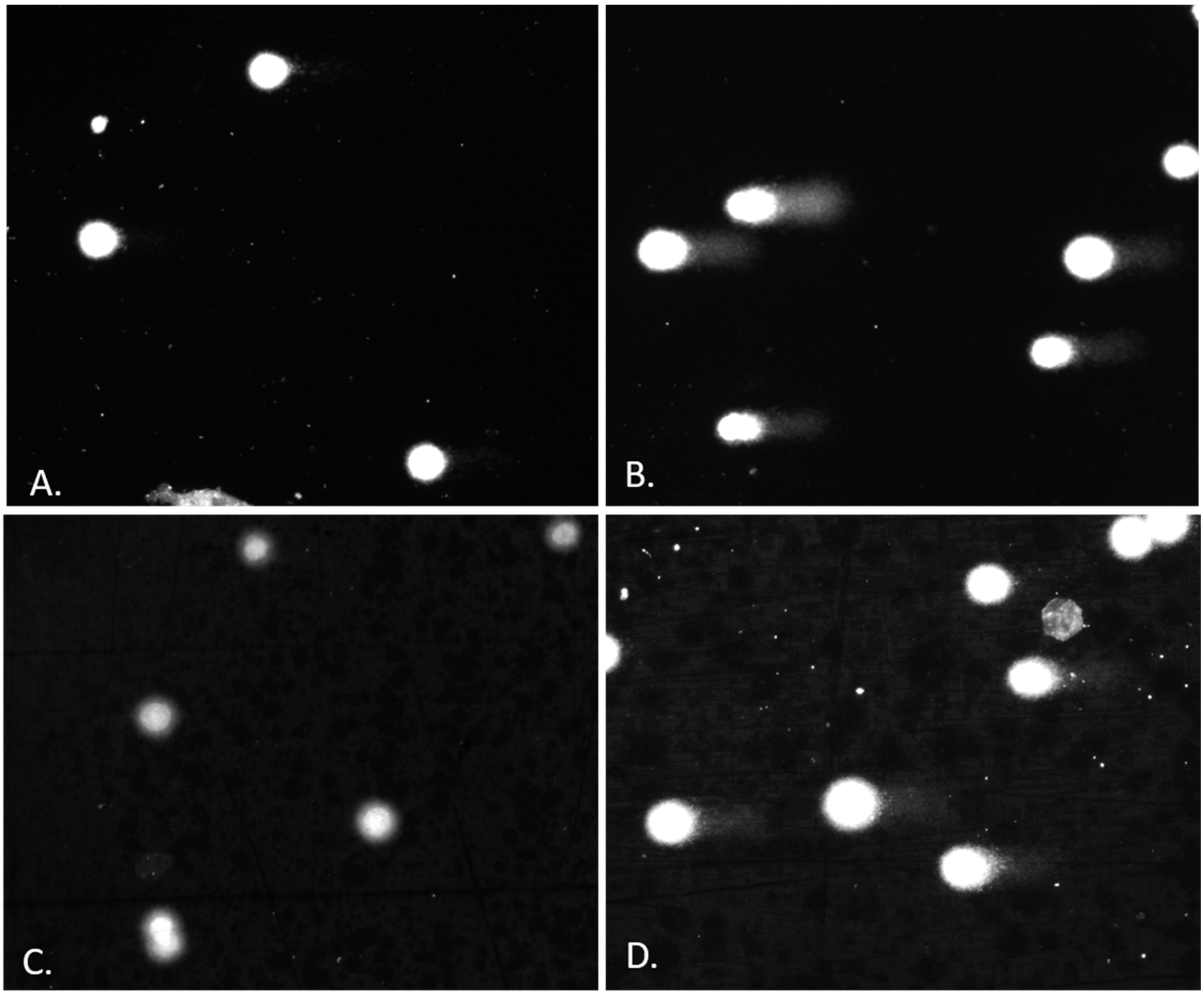

Figure S2. Representative pictures of A549 cells used as substrates in the DNA repair experiment, incubated with placental tissue extracts from healthy control or preeclampsia patients. A) Non exposed (noRo) cells incubated with protein extract of a control placenta; B) Exposed (Ro) cells incubated with protein extract of a control placenta; C) Non exposed (noRo) cells incubated with protein extract of a PE placenta; D) Exposed (Ro) cells incubated with protein extract of a PE placenta.
